# Longitudinal Neuroimaging Reveals Divergent Clinical Associations of Glymphatic Dysfunction and Dopaminergic Degeneration in Parkinson's Disease

**DOI:** 10.1002/hbm.70477

**Published:** 2026-02-23

**Authors:** Taiyuan Liu, Yu Shen, Yan Bai, Suhua Gao, Nan Meng, Wei Wei, Neil Roberts, Meiyun Wang

**Affiliations:** ^1^ Department of Medical Imaging Zhengzhou University People's Hospital & Henan Provincial People's Hospital Zhengzhou China; ^2^ Key Laboratory of Science and Engineering for the Multi‐Modal Prevention and Control of Major Chronic Diseases Ministry of Industry and Information Technology Zhengzhou China; ^3^ Biomedical Research Institute Henan Academy of Sciences Zhengzhou China; ^4^ Translational Research Institute Zhengzhou University People's Hospital, Academy of Medical Science, Zhengzhou University Zhengzhou China; ^5^ The Queen's Medical Research Institute University of Edinburgh Edinburgh UK

**Keywords:** diffusion tensor imaging analysis along the perivascular space, dopamine transporter imaging striatal binding ratio, glymphatic system, Parkinson's disease

## Abstract

To investigate the association between glymphatic function and dopaminergic degeneration in PD assessed via diffusion tensor imaging analysis along the perivascular space (DTI‐ALPS) and dopamine transporter imaging striatal binding ratio (DAT‐SBR), aiming to clarify their controversial relationship and distinct roles in disease progression. A total of 70 early‐stage, drug‐naïve patients with PD and 70 age‐ and sex‐matched healthy controls (HCs) were selected from the Parkinson's Progression Markers Initiative database for cross‐sectional analysis. Longitudinal data at 4‐year follow‐up were available for the PD group. Glymphatic function was evaluated using DTI‐ALPS, and dopaminergic function using DAT‐SBR derived from DAT‐SPECT imaging. Clinical motor and non‐motor assessments were performed at baseline and follow‐up. Correlations between imaging index and clinical variables were analyzed using Spearman correlation and multivariate regression. At baseline, both DTI‐ALPS and DAT‐SBR index were significantly lower in PD patients compared to HCs. Notably, no significant correlation was observed between ALPS and SBR index. Clinically, the DTI‐ALPS index showed negative correlations with body mass index, disease duration, Hoehn and Yahr stage, and UPDRS III scores, and its longitudinal decline correlated with white matter microstructural degeneration. The DAT‐SBR index was negatively correlated with Epworth Sleepiness Scale, REM sleep behavior disorder score, and serum urate. Our findings suggest that glymphatic dysfunction and nigrostriatal denervation represent independent, parallel pathological trajectories in early PD. While the ALPS index may serve as a potential imaging marker of structural network integrity linked to motor execution. These indices offer distinct, complementary mechanistic insights into PD pathology.

## Key Points

- Longitudinal PD cohort: ALPS and DAT‐SBR are reduced vs. controls and decline.
- ALPS and DAT‐SBR show no correlation, indicating distinct pathological pathways.
- ALPS links to motor severity (UPDRS‐III) and white matter structure, serving as a marker of network integrity.

## Graphical abstract

- Both DTI‐ALPS and DAT‐SBR index in patients with PD were significantly lower compared to Healthy Controls, with further reductions observed at the follow‐up.
- Both DTI‐ALPS and DAT‐SBR index in PD showed significant correlation with different clinical measures, while no significant correlation was observed between these two index.

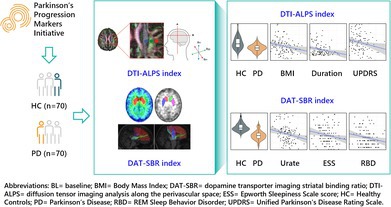

## 1 Introduction

Parkinson's disease (PD) is a progressive neurodegenerative disorder clinically characterized by both motor and non‐motor symptoms (Morris et al. 2024; Poewe et al. 2017). The fundamental pathological basis of PD is the loss of dopaminergic neurons in the substantia nigra pars compacta (Morris et al. 2024). Extensive research indicates that the death of these neurons is primarily caused by the pathological aggregation and accumulation of α‐synuclein, which forms intracellular inclusions known as Lewy bodies (Morris et al. 2024; Pitton Rissardo et al. 2025; Poewe et al. 2017). Although the clinical manifestations and neuronal loss in PD are well documented, the upstream mechanisms leading to α‐synuclein accumulation remain poorly understood. In particular, the role of protein clearance failure in PD pathogenesis is gaining attention, yet the precise mechanisms and their relationship to disease severity and progression are still unclear (Abeliovich and Gitler 2016).

Recent studies have suggested that dysfunction of the glymphatic system—a cerebrospinal fluid (CSF) clearance pathway mediated by aquaporin‐4 (AQP4) water channels on astrocytic endfeet—may contribute to α‐synuclein accumulation and neurodegeneration in PD (Rasmussen et al. 2018; Zou et al. 2019). The glymphatic system functions as a brain‐wide waste‐clearance network, facilitating the paravascular exchange of cerebrospinal and interstitial fluids to eliminate neurotoxic aggregates, most notably amyloid‐β and tau (Sun et al. 2018; Xu et al. 2015). Crucially, glymphatic flow dysfunction has been identified as a cardinal hallmark throughout the PD spectrum, creating a permissive environment for the progressive accumulation of α‐synuclein and subsequent dopaminergic neurodegeneration (Ghaderi et al. 2025; Lv et al. 2025; Zhang et al. 2023; Zhao et al. 2025).

To assess this function non‐invasively, diffusion tensor imaging analysis along the perivascular space (DTI‐ALPS) has emerged as a promising methodology, and has been applied in studies of Alzheimer’s Disease (AD), Amyotrophic Lateral Sclerosis (ALS), and prodromal PD conditions (Liu et al. 2024; Si et al. 2022; Taoka et al. 2017). Utilizing this technique, emerging evidence has identified glymphatic dysfunction as a convergent pathogenic mechanism across proteinopathies (Buccellato et al. 2022; Marecek et al. 2025), with specific observations of flow alterations and perivascular space burdens in patients with both idiopathic and possible REM sleep behavior disorder (RBD) suggesting that such drainage deficits may precede clinical motor symptoms and contribute to early α‐synuclein accumulation (Lee et al. 2022; Marecek et al. 2025; Si et al. 2022, 2020). However, the specific role of ALPS‐index in PD and its association with clinical features remains underexplored.

Conventionally, dopamine transporter (DAT) imaging using single‐photon emission computed tomography (SPECT) offers a well‐established biomarker for assessing nigrostriatal degeneration in PD (Djang et al. 2012; Schwarz et al. 2000). While DAT imaging is sensitive for diagnosing PD and monitoring dopaminergic neuron loss, it provides limited insight into non‐motor symptoms or other underlying pathologies such as impaired protein clearance (Simuni et al. 2018; Weintraub et al. 2005). The interplay between glymphatic failure and dopaminergic denervation remains a critical yet controversial frontier in PD research. Recent investigations integrating DTI‐ALPS index with dopamine transporter imaging striatal binding ratio (DAT‐SBR) have yielded divergent results: while two independent studies by Bae et al. observed no significant correlation between glymphatic flow and nigrostriatal dopaminergic integrity in either PD or RBD cohorts (Bae, Kim, Choi, Choi, et al. 2023; Bae, Kim, Choi, Ryoo, et al. 2023), a more recent study by Marecek et al. demonstrated that reduced ALPS index in PD patients directly correlates with increased nigrostriatal denervation (Marecek et al. 2025). These conflicting findings highlight the complex nature of brain waste clearance and its potential impact on dopaminergic health, emphasizing the urgent need for further investigation within well‐characterized longitudinal cohorts to clarify whether glymphatic dysfunction serves as a primary driver or a secondary consequence of neurodegeneration.

Therefore, the current study aimed to investigate the association between DTI‐ALPS index and DAT‐SBR index in patients with PD and to explore their respective links with motor and non‐motor symptom profiles across disease progression.

## 2 Materials and Methods

### 2.1 Participants

The present study has made use of demographic, clinical and MR imaging data that can be freely downloaded from the website of the Parkinson's Progression Marker Initiative (PPMI) (http://www.ppmi‐info.org/) that was launched more than 10 years ago and is an ongoing multi‐center study with cross‐sectional and longitudinal aspects involving 12 countries that was set up with the aim of identifying markers of the onset and progression of PD. The search terms “PD”, “healthy control”, “age more than 50”, “baseline”, “3T,” “SIEMENS,” “DTI” and “DAT single photon emission computed tomography (SPECT)” were applied to screen the 320 individuals with PD and 73 Healthy Controls (HCs) of the PPMI database.

Of the 320 PD patients initially screened, 250 were excluded due to incomplete longitudinal imaging data (lacking paired DTI or SPECT at 4 years) or image quality issues (including motion artifacts, severe white matter hyperintensities). This resulted in a final cohort of 70 PD patients with complete longitudinal datasets. For the cross‐sectional baseline comparison, we selected 70 age‐ and sex‐matched Healthy Controls (from an initial pool of 73) to ensure statistical comparability with the PD cohort.

Inclusion criteria for patients with PD were: disease duration less than 2 years; early‐stage PD with Hoehn and Yahr stage 1 or 2; drug naïve; and dopamine deficits as assessed by DAT‐SPECT imaging. HCs had no neurological disorders and no first‐degree relatives with PD. Participants with evidence of significant cerebrovascular disease (including stroke, extensive white matter hyperintensities on T2/FLAIR MRI) were excluded from the cohort. The majority of participants in the study were right‐handed. This study was conducted in accordance with the Declaration of Helsinki and was approved by the institutional review boards of each PPMI site. And all participants gave fully informed written consent.

### 2.2 Clinical Assessments

The demographic information that was recorded for the patients with PD who participated in the PPMI study included age, sex, time from diagnosis to enrollment, and number of years of education. A series of scales were applied to evaluate the clinical symptoms that the patients were experiencing and included assessment of both motor and non‐motor function. In particular, the staging of Hoehn and Yahr was used to assess the severity and progression of the disease, and the Movement Disorder Society–Unified Parkinson's Disease Rating Scale (MDS‐UPDRS) was used to evaluate the severity of symptoms and the overall impact of the disease on daily life, especially motor function. Non‐motor symptoms were assessed by using the Rapid Eye Movement Sleep Behavior Disorder Screening Questionnaire (RBDSQ), the Epworth Sleepiness Scale score (ESS) for daytime sleepiness, the University of Pennsylvania Smell Identification Test (UPSIT) score, the Montreal Cognitive Assessment (MoCA) for cognitive function, the Benton Judgment of the Line Orientation Scores (BJLOT) for visuospatial function, the Letter Number Sequencing Score (LNS) and the Symbol Digit Modalities Test (SDMT) for attention and working memory, the Visual Learning and Thinking Animation (VLTANIM) for executive function, and the Hopkins Verbal Learning Test (HVLT) for memory. In addition, the Geriatric Depression Scale Score (GDS) was used for assessing depression, the State‐Trait Anxiety Inventory (STAI) for measuring anxiety (encompassing both state and trait anxiety dimensions), and the Scales for Outcomes in Parkinson's Disease‐Autonomic Dysfunction (SCOPA‐AUT) for evaluating autonomic dysfunction. The concentration of uric acid level in serum was also assessed.

### 2.3 Dopaminergic Imaging

Information regarding how the DAT images were analysed and DAT‐SBR was calculated can be found in the PPMI DatScan SPECT Image Processing manual (https://www.ppmi‐info.org/sites/default/files/docs/PPMI2.0_SPECT_TOM_Final_v6.0_20221201_FE.pdf). Pre‐processing of the DAT images included iterative reconstruction, attenuation correction, spatial normalization, and Gaussian smoothing.

In order to pursue the objectives of the present study, a Region of Interest (ROI) analysis was performed of the SPECT images to obtain the count density for four striatal regions, namely left and right caudate and putamen, as well as for occipital cortex to provide a reference value, and DAT‐SBR was calculated as ((target region/reference region) − 1). To take account of asymmetry, the value of SBR recorded for caudate and putamen refers to whichever side had the lower value.

### 2.4 DTI Image Acquisition and Processing

The baseline and follow‐up DTI images were obtained using a Siemens 3 T MRI system using the following acquisition parameters: Repetition Time (TR) 900 ms, Echo Time (TE) 88 ms, flip angle = 90°, *b*‐value 0 and 1000 s/mm^2^, diffusion gradient directions 30 or 64, voxel size 2 mm × 2 mm × 2 mm or 72 axial sections (http://www.ppmi‐info.org/wp‐content/uploads/2018/02/PPMI‐AM‐13Protocol.pdf for further details).

The FMRIB Software Library 6.0.1 (http://www.fmrib.ox.ac.uk/fsl/) version was used to extend the analysis of the PPMI DTI data. The data were pre‐processed to correct for head movement, adjust the gradient directions to take account of eddy currents and perform brain tissue extraction, and a diffusion tensor model was used to produce a series of diffusion metric images, including color‐coded maps of fractional anisotropy (colFA) together with diffusivity calculated with respect to the direction of the projection (Dxproj, Dyproj, Dzproj) and association fibers (Dxassoc, Dyassoc, Dzassoc).

Subsequently, two experienced neuroradiologists independently placed spherical ROIs (diameter 5 mm) on colFA maps in the projection and association areas at the level of the lateral ventricle body in the left and right cerebral hemispheres. The average of ROI from both hemispheres was utilized. ROIs were meticulously examined on co‐registered FLAIR images to ensure that placement did not coincide with visible white matter hyperintensities. For each ROI, the value of the DTI‐ALPS index was calculated as mean (Dxproj, Dxassoc)/mean (Dyproj, Dzassoc) (Taoka et al. 2017).

To assess white matter microstructural integrity, fractional anisotropy (FA) and mean diffusivity (MD) maps were generated for each participant following diffusion tensor fitting. Tract‐Based Spatial Statistics (TBSS) was then used to enable voxel‐wise group comparisons (Smith et al. 2006). Briefly, individual FA images were nonlinearly registered to standard space, resampled, and used to create a mean FA image, from which a common white matter skeleton was derived and thresholded to represent the centers of major tracts. Each participant's FA data, as well as the corresponding MD data, were subsequently projected onto this skeleton to reduce residual misalignment. Voxel‐wise between‐group inference was performed on the skeletonized FA and MD maps using permutation‐based nonparametric testing with threshold‐free cluster enhancement (TFCE), while controlling for age and sex as covariates in the general linear model (GLM). Statistical significance was assessed at a family‐wise error (FWE) corrected threshold of *p* < 0.05 across the skeleton. The resulting FWE‐significant skeleton clusters were anatomically interpreted by overlaying them on the Johns Hopkins University (JHU) white matter tractography atlas to identify the predominant contributing tracts (Hua et al. 2008). For descriptive reporting and visualization, mean FA/MD values were extracted from the FWE‐significant skeleton mask for each participant and summarized at the group level.

### 2.5 Statistical Analyses

Statistical analyses were performed using IBM SPSS Statistics (version 23, Armonk, NY, USA) and R Software (Version 3.5.3). A *p*‐value of less than 0.05 was used to denote significance.

Continuous variables were assessed for normality using the Shapiro–Wilk test. Variables following a normal distribution were analyzed using parametric tests (*t*‐tests, Pearson correlation); otherwise, non‐parametric tests (Mann–Whitney *U*, Spearman correlation) were employed. In addition, inter‐observer variability between the two readers in respect of the DTI‐ALPS index was assessed using the interclass correlation coefficient (ICC), and one‐way analysis of covariance (ANCOVA) was performed for the DTI‐ALPS index with individual age entered as a covariate (McGraw and Wong 1996). Spearman correlation analysis was performed to investigate the relationship between demographical information, clinical symptom scores, and values of the imaging index in patients with PD.

In the analysis of DTI‐ALPS index, DAT‐SBR index, age, uric acid concentration in serum, and disease duration were the independent variables, and any of MDS‐UPDRS‐III score, ESS score, MoCA score, UPSIT score, BJLOT score, LNS score, SDMT score, VLTANIM score, HVLT score, GDS score, STAI score, SCOPA score, and RBDSQ were the dependent variable. To account for potential confounding effects of age, sex, and BMI on imaging index, additional partial correlation analyses were performed between DTI‐ALPS and DAT‐SBR, adjusting for these covariates. Multivariate linear regression models were constructed to investigate the potential relationship between DTI‐ALPS index and DAT‐SBR index and motor and non‐motor symptoms in the patients with PD. The results were presented as standardized β coefficients.

The patients with PD were divided into several sub‐groups, namely (1) Hoehn and Yahr stage 1 and stage 2; (2) patients with GDS ≥ 5 were considered “Depressed” [PD‐D] and with GDS < 5 “Not Depressed” [PD‐ND]; (3) patients with MoCA score < 24 were considered cognitively impaired PD [PDCI] and with MoCA score ≥ 24 to have normal cognition [PDN]; (4) patients with ESS score ≥ 10 were considered to have excessive daytime sleepiness [PD‐WE] and with ESS score < 10 to be without excessive daytime sleepiness [PD‐WOE]. Potential differences in the value of the DTI‐ALPS index between these sub‐groups were investigated.

In the longitudinal study, clinical assessment scores and the index derived from imaging were compared between baseline (PD‐BL) and follow‐up (PD‐F) using paired *t*‐tests, Pearson's chi‐square tests and non‐parametric tests. The rate of change of a variable Δ/*T* was calculated as ([value at follow‐up − value at baseline]/time interval (months)) and expressed as a percentage (Wu et al. 2020). Linear regression analyses were performed to investigate whether correlations potentially existed between DTI‐ALPS index, DAT‐SBR index and clinical symptoms.

## 3 Results

### 3.1 Demographic Information and Clinical Assessment Scores

In the cross‐sectional study, 70 PD patients and 70 age‐ and sex‐matched HCs at baseline were included in the PPMI database. The demographic characteristics and clinical information for the 70 patients with PD and 70 HCs are presented in Table 1.

**TABLE 1:** Demographics and clinical characteristics and imaging findings of cross‐sectional participants.

	Healthy control (*n* = 70)	PD (*n* = 70)	*p*
Clinical variables			
Sex (F/M)	31/39	31/39	1.00
Age, years	64.37 ± 7.67	64.74 ± 7.93	0.797
Education, years	16.13 ± 3.45	15.32 ± 2.79	0.157
BMI	26.29 ± 4.62	27.69 ± 5.22	0.128
Duration, month (IQR)	NA	8.25 (4.19, 16.53)	NA
Total MDS‐UPDRS	3.81 ± 4.35	33.80 ± 12.80	< 0.001
Hoehn & Yahr (IQR)	NA	1 (1, 2)	NA
MoCA	27.95 ± 1.44	26.78 ± 2.46	0.002
UPSIT	32.31 ± 5.44	23.00 ± 8.82	< 0.001
RBDSQ	2.32 ± 2.28	3.93 ± 2.72	0.001
ESS	5.36 ± 3.28	5.75 ± 3.18	0.507
BJLOT	13.22 ± 1.78	12.72 ± 2.03	0.152
LNS	10.90 ± 2.33	10.38 ± 2.85	0.283
SDMT	46.10 ± 9.48	41.07 ± 8.15	0.002
GDS	1.56 ± 2.87	2.12 ± 2.09	0.228
STAI	55.76 ± 14.37	61.28 ± 16.86	0.057
SCOPA	6.42 ± 3.93	10.13 ± 6.59	< 0.001
VLTANIM	22.83 ± 4.90	21.47 ± 4.99	0.135
Urate	5.46 ± 1.47	5.03 ± 1.41	0.115
Imaging parameters			
ALPS	1.62 ± 0.42	1.41 ± 0.13	< 0.001
SBR	2.44 ± 0.47	1.54 ± 0.47	< 0.001

Abbreviations: ALPS, analysis along the perivascular space; BJLOT, Benton Judgment of the Line Orientation Scores; BMI, body mass index; ESS, Epworth Sleepiness Scale score; F, female; GDS, Geriatric Depression Scale; IQR, interquartile range; LNS, Letter Number Sequencing Score; M, male; MDS‐UPDRS, Movement Disorder Society‐Unified Parkinson's Disease Rating Scale; MoCA, Montreal Cognitive Assessment; NA, not available; PD, Parkinson's disease; RBDSQ, Rapid Eye Movement Sleep Behavior Disorder Screening Questionnaire; SBR, specific binding ratio; SCOPA, Scales for Outcomes in Parkinson's Disease; SDMT, Symbol Digit Modalities Test; STAI, State–Trait Anxiety Inventory (encompassing both state and trait anxiety dimensions); UPSIT, University of Pennsylvania Smell Identification Test; VLTANIM, Visual Learning and Thinking Animation.

Value 0.898 (confidence interval: 0.882–0.911, *p* < 0.001) obtained for ICC indicates there is excellent agreement between the two observers in obtaining the value of the DTI‐ALPS index.

Patients with PD showed no statistically significant differences in age, sex, years of education or BMI in comparison with HCs. However, the two groups differed significantly in respect of scores on MDS‐UPDRS (*p* < 0.001), UPSIT (*p* < 0.001), MoCA (*p* = 0.002), RBDSQ (*p* = 0.001), SDMT (*p* = 0.002) and SCOPA (*p* < 0.001).

Follow‐up data were available for 70 of the patients with PD. The changes in clinical characteristics of the 70 patients at baseline (i.e., PD‐BL) and at follow‐up (i.e., PD‐F) are summarized in Table 2.

**TABLE 2:** Demographics of the follow‐up participants.

	PD‐BL (*n* = 70)	PD‐F (*n* = 70)	*p*
Clinical variables			
Sex (F/M)	28/42	28/42	1.00
Age, years	63.34 ± 7.15	67.39 ± 7.15	< 0.001
Education, years	15.01 ± 3.10	15.01 ± 3.10	1.00
BMI	27.10 ± 4.43	26.74 ± 4.23	0.125
MoCA (IQR)	28 (26, 29)	28 (26, 29)	0.489
UPSIT	20.34 ± 8.67	NA	NA
RBDSQ	4.03 ± 2.60	4.52 ± 3.11	0.042
ESS	6.44 ± 3.50	7.62 ± 4.91	0.023
BJLOT	12.55 ± 2.18	12.73 ± 2.09	0.635
LNS	10.28 ± 2.86	9.83 ± 3.20	0.154
SDMT	40.21 ± 9.00	37.77 ± 10.68	0.011
GDS (IQR)	2 (0, 3)	1 (1, 3)	0.931
STAI	66.06 ± 18.00	63.73 ± 16.13	0.106
SCOPA	9.26 ± 5.81	12.30 ± 6.53	< 0.001
VLTANIM	21.69 ± 4.51	22.03 ± 6.13	0.780
Urate	5.32 ± 1.35	5.15 ± 1.23	0.088
Imaging findings			
ALPS	1.59 ± 0.27	1.53 ± 0.33	0.042
SBR	1.34 ± 0.38	0.99 ± 0.39	< 0.001

Abbreviations: ALPS, analysis along the perivascular space; BJLOT, Benton Judgment of the Line Orientation Scores; BMI, body mass index; ESS, Epworth Sleepiness Scale score; F, female; GDS, Geriatric Depression Scale; LNS, Letter Number Sequencing Score; M, male; MoCA, Montreal Cognitive Assessment; NA, not available; PD‐BL, Parkinson's disease at baseline; PD‐F, Parkinson's disease at follow‐up; RBDSQ, Rapid Eye Movement Sleep Behavior Disorder Screening Questionnaire; SBR, striatal binding ratio; SCOPA, Scales for Outcomes in Parkinson's Disease; SDMT, Symbol Digit Modalities Test; STAI, State–Trait Anxiety Inventory (encompassing both state and trait anxiety dimensions); UPSIT, University of Pennsylvania Smell Identification Test; VLTANIM, Visual Learning and Thinking Animation.

In particular, the results of paired *t*‐tests revealed significant differences in RBDSQ (PD‐BL < PD‐F, *p* = 0.042), ESS (PD‐BL < PD‐F, *p* = 0.023), SDMT (PD‐BL > PD‐F, *p* = 0.011), SCOPA (PD‐BL < PD‐F, *p* < 0.001) between the two groups.

### 3.2 Relationship Between DTI‐ALPS and DAT‐SBR and Clinical Assessment Scores

**FIGURE 1:**
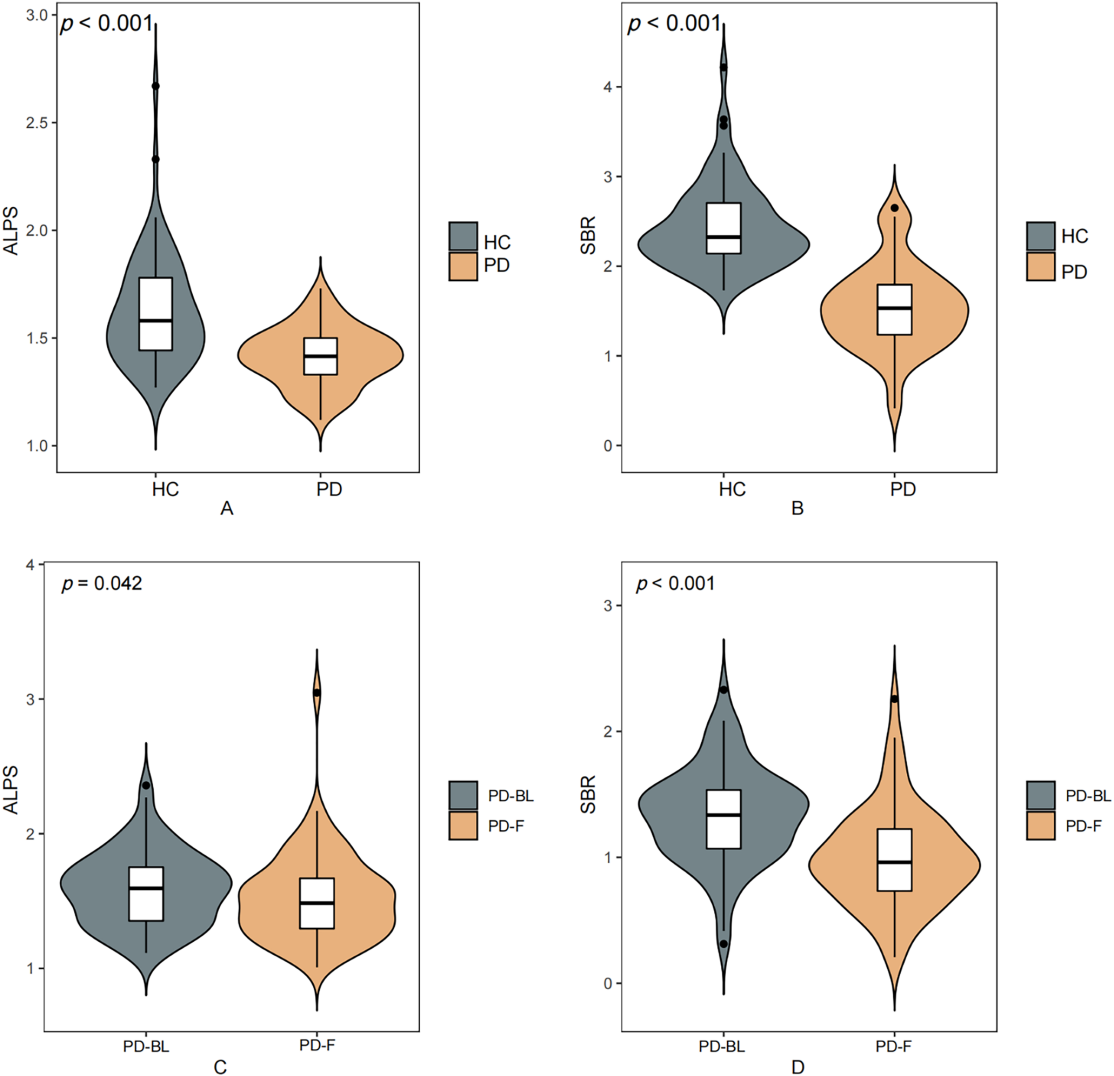
A Box and whisker plot of the data from DTI‐ALPS index between healthy control and PD group in the cross‐sectional study. B Box and whisker plot of the data from DAT‐SBR index between healthy control and PD group in the cross‐sectional study. C Box and whisker plot of the data from DTI‐ALPS index between PD‐BL group and PD‐F group in the longitudinal study. D Box and whisker plot of the data from DAT‐SBR index between PD‐BL group and PD‐F group in the longitudinal study. The data are represented as box plots, where the box signifies the median and interquartile range, and the whiskers indicate the maximum and minimum values within the dataset. ALPS, analysis along the perivascular space; SBR, striatal binding ratio; HC, healthy control; PD, Parkinson's disease; PD‐BL, Parkinson's disease at baseline; PD‐F, Parkinson's disease at follow‐up.

**FIGURE 2:**
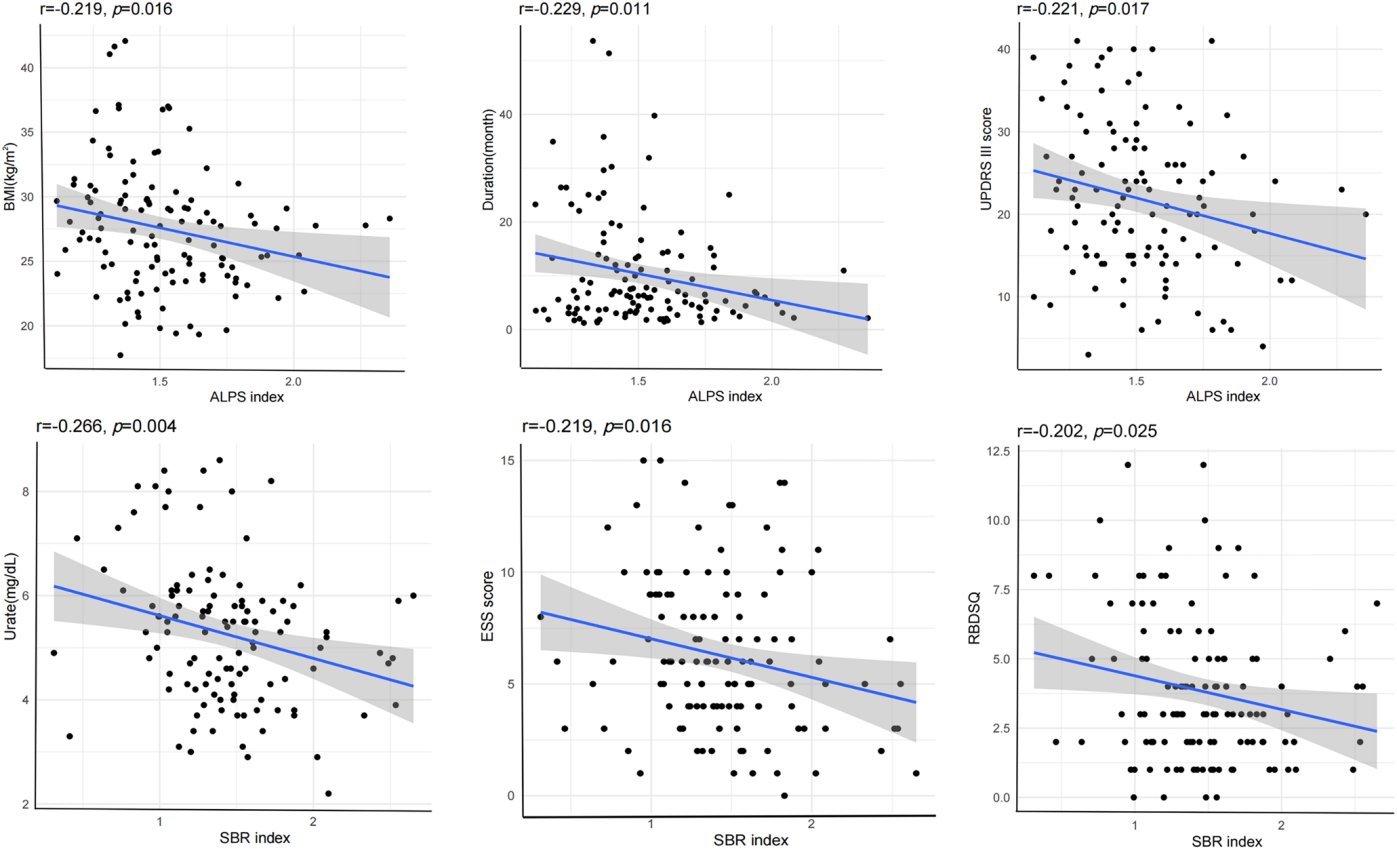
Correlation between DTI‐ALPS index and DAT‐SBR index and clinical features in patients with PD in the cross‐sectional study. Each data point represents the measurements of an individual participant. The solid line represents the curve fitted through correlation analysis. ALPS, analysis along the perivascular space; SBR, striatal binding ratio; BMI, body mass index; UPDRS III, Unified Parkinson's Disease Rating Scale part III; ESS, Epworth Sleepiness Scale score; RBDSQ, Rapid Eye Movement Sleep Behavior Disorder Screening Questionnaire.

At baseline, the value of the DTI‐ALPS index and DAT‐SBR index (1.62 ± 0.42 vs. 1.41 ± 0.13, *p* < 0.001; 2.44 ± 0.47 vs. 1.54 ± 0.47, *p* < 0.001) were lower for the patients with PD than the HCs (Figure 1). Furthermore, for the patients with PD Spearman correlation analysis revealed negative correlations between DTI‐ALPS index and years of education (*r* = −0.349, *p* < 0.001), BMI (*r* = −0.219, *p* = 0.016), duration (*r* = −0.229, *p* = 0.011), Hoeh and Yahr stage (*r* = −0.246, *p* = 0.008) and UPDRS III (*r* = −0.221, *p* = 0.017), while the value of the DAT‐SBR index was negatively correlated with ESS (*r* = −0.219, *p* = 0.016), RBDSQ (*r* = −0.202, *p* = 0.025), uric acid concentration in plasma (*r* = −0.266, *p* = 0.004) and positively correlated with SDMTOTAL (*r* = 0.222, *p* = 0.014) (Figure 2 and Table 3).

**TABLE 3:** Correlation analysis between imaging characteristics and clinical features in PD.

	SBR		ALPS		△SBR/T		△ALPS/T	
	*r*/*z*	*p*	*r*/*z*	*p*	*r*/*z*	*p*	*r*/*z*	*p*
Age	−0.004	0.967	−0.069	0.447	−0.172	0.155	−0.108	0.372
EDUCYRS	0.063	0.491	−0.349^b^	< 0.001	−0.191	0.114	−0.102	0.399
BMI	−0.004	0.967	−0.219^a^	0.016	−0.080	0.513	0.022	0.857
Duration	0.013	0.884	−0.229^a^	0.011	−0.043	0.721	−0.019	0.873
UPSIT	0.085	0.387	−0.144	0.142	0.135	0.266	0.182	0.131
MoCA	−0.018	0.847	0.039	0.669	0.114	0.347	−0.177	0.143
BJLOT	0.097	0.291	−0.062	0.496	0.006	0.961	−0.092	0.447
LNS	0.083	0.362	−0.048	0.596	0.090	0.459	−0.098	0.421
SDMT	0.222^a^	0.014	0.005	0.953	0.055	0.648	−0.010	0.935
VLTANIM	0.020	0.825	−0.074	0.420	0.132	0.277	0.271^a^	0.023
ESS	−0.219^a^	0.016	0.097	0.291	−0.100	0.410	0.041	0.735
RBDSQ	−0.202^a^	0.025	0.054	0.552	−0.180	0.136	0.059	0.627
GDS	−0.070	0.442	0.001	0.987	−0.021	0.860	−0.008	0.947
STAI	−0.107	0.239	0.124	0.174	0.001	0.997	−0.196	0.104
SCOPA	−0.163	0.075	−0.011	0.906	0.139	0.257	−0.092	0.454
Hoehn & Yahr	−0.038	0.683	−0.246^b^	0.008	0.038	0.756	−0.204	0.091
UPDRS III	−0.097	0.301	−0.221^a^	0.017	−0.021	0.861	−0.051	0.672
Urate	−0.266^b^	0.004	−0.058	0.534	−0.073	0.549	0.062	0.612
SBR	NA	NA	−0.067	0.464	NA	NA	NA	NA

Abbreviations: ALPS, analysis along the perivascular space; BJLOT, Benton Judgment of the Line Orientation Scores; BMI, body mass index; EDUCYRS, education years; ESS, Epworth Sleepiness Scale score; GDS, Geriatric Depression Scale; LNS, Letter Number Sequencing Score; MoCA, Montreal Cognitive Assessment; NA, not available; PD, Parkinson's disease; RBDSQ, Rapid Eye Movement Sleep Behavior Disorder Screening Questionnaire; SBR, striatal binding ratio; SCOPA, Scales for Outcomes in Parkinson's Disease; SDMT, Symbol Digit Modalities Test; STAI, State‐Trait Anxiety Inventory (encompassing both state and trait anxiety dimensions); UPDRS, Unified Parkinson's Disease Rating Scale; UPSIT, University of Pennsylvania Smell Identification Test; VLTANIM, Visual Learning and Thinking Animation.

^a^ At the 0.05 level (two‐tailed), the correlation was significant.

^b^ At the 0.01 level (two‐tailed), the correlation was significant.

After controlling for age, sex, and BMI, the correlation between DAT‐SBR index and DTI−ALPS index was also investigated and was found to be of no statistical significance (*r* = −0.121, *p* = 0.195).

For the multivariate analysis, in the patients with PD, the independent variables of age, duration, uric acid concentration in serum, DAT‐SBR index, and DTI‐ALPS index were all significantly associated with the dependent variables of LNS (*F* = 3.239, *p* = 0.009, *R* ^2^ = 0.128), SDMTOTAL (*F* = 6.367, *p* < 0.001, *R* ^2^ = 0.224), VLTANIM (*F* = 5.006, *p* < 0.001, *R* ^2^ = 0.185), SCOPA (*F* = 4.688, *p* = 0.001, *R* ^2^ = 0.178), UPDRS III (*F* = 2.541, *p* = 0.033, *R* ^2^ = 0.109) (Table 4).

**TABLE 4:** Multivariate linear regression analysis.

	Model characteristics			ALPS		SBR		Age		Duration		Urate	
	*F*	*p*	*R* ^2^	*β*	*p*	*β*	*p*	*β*	*p*	*β*	*p*	*β*	*p*
UPSIT	1.981	0.089	0.095	−0.161	0.111	0.050	0.629	−0.168	0.100	0.013	0.898	−0.167	0.121
MoCA	1.144	0.342	0.049	0.037	0.697	0.009	0.927	−0.185	0.053	0.112	0.244	−0.048	0.627
BJLOT	2.067	0.075	0.087	−0.097	0.308	0.101	0.290	−0.187	0.046	−0.200	0.036	0.033	0.734
LNS	3.239	0.009	0.128	−0.040	0.667	0.088	0.343	−0.273	0.003	0.166	0.073	−0.097	0.305
SDMT	6.367	< 0.001	0.224	0.013	0.878	0.196	0.027	−0.319	< 0.001	0.142	0.103	−0.184	0.040
VLTANIM	5.006	< 0.001	0.185	−0.077	0.386	0.005	0.954	−0.410	< 0.001	0.135	0.129	0.004	0.966
ESS	2.043	0.078	0.086	0.084	0.377	−0.250	0.009	0.071	0.447	−0.002	0.983	0.051	0.594
RBDSQ	1.180	0.323	0.051	0.066	0.493	−0.190	0.051	0.025	0.793	0.077	0.420	0.035	0.720
GDS	0.097	0.993	0.004	0.005	0.959	0.012	0.900	0.007	0.942	0.003	0.979	0.067	0.505
STAI	0.702	0.623	0.031	0.097	0.316	−0.070	0.476	−0.088	0.358	−0.044	0.649	−0.049	0.621
SCOPA	4.688	0.001	0.178	0.059	0.516	−0.136	0.140	0.162	0.071	0.313	0.001	0.122	0.187
UPDRS III	2.541	0.033	0.109	−0.188	0.051	−0.095	0.327	0.112	0.239	0.178	0.066	0.022	0.819
Hoehn & Yahr	2.215	0.058	0.096	−0.223	0.022	−0.039	0.684	0.126	0.187	0.111	0.252	0.006	0.950

Abbreviations: ALPS, analysis along the perivascular space; BJLOT, Benton Judgment of the Line Orientation Scores; ESS, Epworth Sleepiness Scale score; GDS, Geriatric Depression Scale; LNS, Letter Number Sequencing Score; MoCA, Montreal Cognitive Assessment; PD, Parkinson's Disease; RBDSQ, Rapid Eye Movement Sleep Behavior Disorder Screening Questionnaire; SBR, striatal binding ratio; SCOPA, Scales for Outcomes in Parkinson's Disease; SDMT, Symbol Digit Modalities Test; STAI, State–Trait Anxiety Inventory (encompassing both state and trait anxiety dimensions); UPDRS, Unified Parkinson's Disease Rating Scale; UPSIT, University of Pennsylvania Smell Identification Test; VLTANIM, Visual Learning and Thinking Animation.

The sub‐group analysis revealed no significant differences between PD‐D and PD‐ND, PD at stage 1 and stage 2, PD‐CI and PDN, and PD‐WE and PD‐WOE (Table S1).

### 3.3 Follow‐Up of DTI‐ALPS and DAT‐SBR Index

With regard to follow‐up, the value of DTI‐ALPS index and DAT‐SBR index were lower for PD‐F compared to PD‐BL (1.59 ± 0.27 vs. 1.53 ± 0.33, *p* = 0.042; 1.34 ± 0.38 vs. 0.99 ± 0.39, *p* < 0.001) (Figure 1 and Table 2).

Spearman correlation analysis revealed no significant correlations between △DAT‐SBR index/T and clinical characteristics. However, △DTI‐ALPS index/T was positively correlated with VLTANIM score (*r* = 0.271, *p* = 0.023).

For the multi‐variate analysis, age, duration, uric acid concentration in plasma, DAT‐SBR index and DTI‐ALPS index at baseline showed no significant correlation with ΔDAT‐SBR index/T (*F* = 0.721, *p* = 0.610, *R* ^2^ = 0.062) or △DTI‐ALPS index/T (*F* = 1.033, *p* = 0.407, *R* ^2^ = 0.086) (Table S2).

### 3.4 Between‐Group Differences in White Matter Microstructural Measures

In the cross‐sectional analysis, participants with PD exhibited decreased MD throughout the white matter skeleton compared to matched controls. Specifically, affected regions included the corticospinal tract, anterior thalamic radiation, uncinate fasciculus, and the genu of the corpus callosum. However, no significant differences were observed in FA values (Figure S1).

In the longitudinal analysis, compared to baseline, the PD group showed reduced FA in the corticospinal tract, anterior thalamic radiation, and corpus callosum, alongside elevated MD in the inferior longitudinal fasciculus, superior longitudinal fasciculus, corticospinal tract, and the genu of the corpus callosum (Figure S2).

After controlling for age and sex, partial correlation analysis revealed that the ΔDTI‐ALPS index/T was positively correlated with ΔMD in regions including the bilateral anterior thalamic radiation, right corpus callosum, left cingulum (hippocampus), bilateral corticospinal tracts, left inferior fronto‐occipital fasciculus, and left inferior longitudinal fasciculus. Conversely, it was negatively correlated with ΔFA in the bilateral anterior thalamic radiation, bilateral cingulum (cingulate gyrus and hippocampus), bilateral corticospinal tracts, bilateral inferior fronto‐occipital fasciculus, bilateral inferior longitudinal fasciculus, bilateral superior longitudinal fasciculus, left superior longitudinal fasciculus (temporal part), and left uncinate fasciculus (Tables S3 and S4).

Linear regression analyses further confirmed that the ΔDTI‐ALPS index/T was positively associated with ΔMD in the left anterior thalamic radiation, bilateral corticospinal tracts, right corpus callosum, left inferior fronto‐occipital fasciculus, and left inferior longitudinal fasciculus. Furthermore, a negative association was found with ΔFA in regions including the bilateral anterior thalamic radiation, bilateral corpus callosum, bilateral cingulum (cingulate gyrus and hippocampus), bilateral corticospinal tracts, bilateral inferior fronto‐occipital fasciculus, bilateral inferior longitudinal fasciculus, left superior longitudinal fasciculus (including the temporal part), and left uncinate fasciculus (Tables S5 and S6).

## 4 Discussion

In this study, we observed that both the DTI‐ALPS index and the DAT‐SBR index were significantly reduced in patients with PD compared to healthy controls, with both indices showing a progressive decline over a four‐year follow‐up. Importantly, the DTI‐ALPS index was negatively associated with body mass index, disease duration, Hoehn and Yahr stage, and motor severity scores (UPDRS III), while the DAT‐SBR index was correlated with sleep‐related disturbances and serum urate levels. Notably, while both systems exhibited impairment, no significant correlation was observed between ALPS and SBR at baseline. This lack of association, coupled with their divergent longitudinal correlations—such as the correlation between SBR and serum urate versus the link between ALPS and motor severity—suggests that nigrostriatal dopaminergic denervation and neurofluid transport alterations may represent independent but parallel pathological processes in early PD.

Our findings of an uncoupled relationship between ALPS‐index and DAT‐SBR align with recent evidence from large‐scale cohorts of both PD and REM sleep behavior disorder. Specifically, Bae et al. observed that while glymphatic flow is impaired in PD (Bae, Kim, Choi, Choi, et al. 2023), it does not directly correlate with striatal dopamine transporter binding levels. This observation was further extended to prodromal stages, where no significant association was detected between glymphatic clearance and dopaminergic integrity in iRBD patients (Bae, Kim, Choi, Ryoo, et al. 2023). Collectively, these data, including those from our refined longitudinal cohort, suggest that glymphatic dysfunction and nigrostriatal denervation may follow independent or non‐linear pathological trajectories during the early phases of neurodegeneration.

Notably, Marecek et al. found that correlations with the caudate were only detectable via automated ROI selection, a finding not replicated by their manual methods (Marecek et al. 2025). In contrast, our study utilized expert‐guided manual placement with a stringent exclusion of white matter hyperintensities (WMH) through co‐registered FLAIR imaging. By ensuring ROI integrity and focusing on the SBR of the more severely affected hemisphere, our findings may more accurately reflect the early‐stage, non‐linear relationship between glymphatic failure and dopaminergic loss. These results suggest that while both systems are compromised in PD, they may follow independent temporal trajectories in the disease's initial phases.

The DAT‐SBR index was significantly reduced in patients with PD at both baseline and follow‐up, reaffirming its utility as a marker of dopaminergic degeneration (Djang et al. 2012; Schwarz et al. 2000). Notably, DAT‐SBR was inversely associated with sleep disturbance scores (ESS and RBDSQ), suggesting that dopaminergic dysfunction contributes to the common sleep‐related symptoms in PD (Stefani and Hogl 2020).

Our baseline analysis revealed a significant negative correlation between serum urate and DAT‐SBR in early‐stage, drug‐naive PD patients (*r* = −0.266, *p* = 0.004), evaluated prior to the initiation of dopaminergic therapy and therefore not subject to ON/OFF medication state effects as defined in the PPMI protocol (Simuni et al. 2018). This inverse relationship likely represents a compensatory upregulation of systemic antioxidant defenses in response to intense nigrostriatal oxidative stress (Haryuni et al. 2024). Thus, higher baseline urate levels may serve as a marker of greater oxidative burden and more severe dopaminergic denervation at this stage, potentially overshadowing its putative neuroprotective effects (Hasimoglu et al. 2020; Seifar et al. 2022). In line with these findings, our longitudinal analysis showed that baseline urate did not significantly predict the rate of dopaminergic decline (ΔSBR/T: *β* = −0.141, *p* = 0.330). The negative coefficient suggests that higher urate trends with faster neurodegeneration, reinforcing the baseline observation that elevated urate is associated with “worse” imaging outcomes. The lack of statistical significance indicates that urate is not a primary driver of disease trajectory, consistent with the negative results of the SURE‐PD3 clinical trial (Parkinson Study Group et al. 2021). Furthermore, the divergent trends of urate on ΔSBR/T (negative) versus ΔALPS/T (*β* = 0.038, *p* = 0.788) support our core finding that dopaminergic degeneration and glymphatic dysfunction evolve as relatively independent pathological processes. Finally, as a systemic metabolic marker subject to high individual variability, serum urate may have limited sensitivity in reflecting local nigral pathology (Hasimoglu et al. 2020; Parnetti et al. 2019; Seifar et al. 2022).

Considering that our findings are derived from a refined cohort of 70 PD patients, they may capture the complex non‐linear relationship between structural denervation and functional impairment characteristic of early disease stages. Consequently, this study did not observe the significant but weak correlation between DAT‐SBR and UPDRS‐III scores reported in larger populations (Simuni et al. 2018). However, our results are fundamentally aligned with the longitudinal evidence from Simuni et al. (Simuni et al. 2018), who demonstrated that the rate of change in dopaminergic binding does not correlate with the rate of clinical motor progression. This lack of association is further supported by our asymmetric analysis—using the SBR of the more severely affected hemisphere—which suggests that motor severity in early PD is likely influenced by compensatory neural mechanisms rather than a direct linear decline in striatal dopamine transporters.

Our findings of significantly reduced ALPS index in patients with PD align with an expanding body of clinical evidence documenting glymphatic impairment throughout the PD spectrum. This observation is robustly supported by a recent large‐scale systematic review and meta‐analysis, which confirmed that glymphatic flow dysfunction is a cardinal hallmark of the disease (Ghaderi et al. 2025). Furthermore, these results are consistent with pioneering DTI‐ALPS studies and recent investigations that have collectively established a clear link between impaired paravascular drainage and the progression of synucleinopathy (Ghaderi et al. 2025; Lv et al. 2025; Zhao et al. 2025). Beyond the intrinsic pathology of PD, we also identified body composition as a potential contributor to glymphatic insufficiency. The negative correlation we observed between DTI‐ALPS and BMI is consistent with recent reports linking higher BMI to impaired glymphatic function in PD (Tian et al. 2024). This association may be partly mediated by comorbid conditions such as obstructive sleep apnea (OSA), which is more prevalent in individuals with higher BMI and has itself been linked to glymphatic dysfunction in early PD (Nepozitek et al. 2025; Nepozitek and Sonka 2025).

Adhering to the refined methodological framework proposed by Taoka et al., we interpret the DTI‐ALPS index as a metric of directional water diffusivity along the perivascular space, rather than a direct quantification of glymphatic flow (Taoka et al. 2024). This distinct physical basis likely explains why the ALPS index demonstrated superior sensitivity in reflecting clinical motor severity compared to dopaminergic imaging in our early‐stage cohort. While baseline DAT‐SBR showed no significant correlation with motor symptoms (UPDRS‐III: *r* = −0.097, *p* = 0.301), the ALPS index exhibited a significant negative correlation (*r* = −0.221, *p* = 0.017). This dissociation suggests that the ALPS index captures more than just fluid dynamics. As it is derived from diffusivity within projection and association fibers, it inherently reflects the white matter microstructural integrity of the motor network (Schilling et al. 2025). Since efficient motor execution relies on signal transmission through white matter pathways such as the corticospinal tract, a reduced ALPS index may signify microstructural damage that impedes neural connectivity, thereby directly exacerbating bradykinesia (Schilling et al. 2025).

To further isolate these mechanisms, we incorporated complementary diffusion metrics, specifically FA and mean diffusivity (MD). The longitudinal data revealed that ALPS index changes correlated with shifts in FA and MD. This suggests that in PD, the ALPS index reflects a multifaceted interplay between paravascular fluid dynamics and progressive white matter degeneration, distinguishing it from conditions like metabolic syndrome where ALPS reduction appears driven primarily by perivenous water diffusivity (Andica et al. 2023). These divergent findings underscore the disease‐specific nature of neurofluid alterations and the necessity of standardized white matter evaluations across different pathologies.

This study has several limitations. The DTI‐ALPS index reflects diffusivity near the lateral ventricles and may not capture changes in basal ganglia regions directly involved in dopaminergic signaling. Furthermore, the PPMI dataset comprises early‐stage PD patients, limiting the generalizability of findings to more advanced disease stages. As an exploratory analysis, this study utilized uncorrected statistical thresholds to reduce the likelihood of Type II errors. Consequently, the results may be more susceptible to false positives and should be considered preliminary, requiring replication in future confirmatory studies. Finally, while both indices provide useful biomarkers, further interventional or longitudinal studies are necessary to delineate causal relationships and explore therapeutic modulation of the glymphatic pathway in PD (Rezai et al. 2024).

## 5 Conclusions

This longitudinal study demonstrates that the reduction of the DTI‐ALPS index and nigrostriatal dopaminergic degeneration represent uncoupled, parallel pathological trajectories in early PD. Adhering to a refined physical interpretation (Taoka et al. 2024), the ALPS index correlated with motor severity and BMI, serving as a marker of white matter microstructural integrity necessary for motor execution. In contrast, DAT‐SBR correlated with sleep disturbances and serum urate, reflecting the systemic metabolic and oxidative burden of the disease. These findings support the use of ALPS and SBR as complementary biomarkers—targeting structural connectivity versus neurochemical degeneration—to comprehensively capture the multifaceted pathology of PD.

## Author Contributions

M.W. contributed to the conception of the work. T.L. and Y.S. contributed to the design of the work. Y.S., N.M., and W.W. contributed to the acquisition and analysis of image data for the work. Y.B. and S.G. contributed to the interpretation of data for the work. T.L. contributed significantly to drafting the manuscript. N.R. and M.W. contributed to revising it critically for important intellectual content. All authors have read and approved the final version of the manuscript and agree to be accountable for all aspects of the work.

## Funding

This research was supported by National Natural Science Foundation of China (82471963); Medical Science and Technology Research Project of Henan Province (SBGJ202303007, LHGJ20210028, SBGJ202103007); Open Project of Key Laboratory of Science and Engineering for the Multi‐modal Prevention and Control of Major Chronic Diseases Ministry of Industry and Information Technology (MCD‐2023‐1‐17); Special Training Program for Clinical Research Physicians under the “Three 100” Initiative of Henan Province (HNCRD202406); Science and Technology Project of Henan Province (242102311029).

## Ethics Statement

This study was conducted in accordance with the Declaration of Helsinki and was approved by the institutional review boards of each PPMI site.

## Consent

All participants gave fully informed written consent.

## Conflicts of Interest

The authors declare no conflicts of interest.

## Supporting information


**Data S1:** Supporting Information.

## Data Availability

The data supporting the findings of this study are available from the Parkinson's Progression Markers Initiative (PPMI) database (www.ppmi‐info.org/access‐dataspecimens/download‐data).
