# Supplementary material for: Longitudinal Neuroimaging Reveals Divergent Clinical Associations of Glymphatic Dysfunction and Dopaminergic Degeneration in Parkinson's Disease
Source: Hum Brain Mapp. 2026 Feb 23;47(3):e70477. doi: 10.1002/hbm.70477 (PMC12929184; doi:10.1002/hbm.70477)

**Supplementary files**

**Table S1** Subgroup analysis of ALPS-index of PD patients.

|  | **Post hoc tests (*p*-value)** | | | |
| --- | --- | --- | --- | --- |
|  | **PD-D(7) vs PD-ND(62)** | **PD at stage 1(24) vs stage 2(40)** | **PD-CI(11) vs PDN(58)** | **PD-WE(9) vs PD-WOE(60)** |
| **ALPS** | 0.269 | 0.318 | 0.482 | 0.175 |

**Note:** ALPS, analysis along the perivascular space; According to GDS score (Participants with GDS ≥ 5 are “Depressed” [PD-D], Participants with GDS < 5 are “Not Depressed”[PD-ND] ;according to MoCA scores (cognitively impaired PD [PDCI]: MoCA score <24; and PD with normal cognition [PDN]: MoCA score ≥24; according to ESS score (PD with excessive daytime sleepiness[PD-WE]: ESS score ≥10 and PD without excessive daytime sleepiness[PD-WOE]: ESS score <10.)

**Table S2** Multivariate linear regression models in the Longitudinal study.

|  | **Model characteristics** | | | **ALPS (baseline)** | | **SBR(baseline)** | | **Age** | | **Duration** | | **Urate** | |
| --- | --- | --- | --- | --- | --- | --- | --- | --- | --- | --- | --- | --- | --- |
|  | F | *p* | R^2^ | β | *p* | β | *p* | β | *p* | β | *p* | β | *p* |
| **ΔALPS /T** | 1.033 | 0.407 | 0.086 | -0.249 | 0.062 | 0.050 | 0.707 | -0.135 | 0.329 | -0.111 | 0.411 | 0.038 | 0.788 |
| **ΔSBR/T** | 0.721 | 0.610 | 0.062 | 0.006 | 0.966 | -0.214 | 0.119 | -0.083 | 0.552 | 0.009 | 0.946 | -0.141 | 0.330 |

**Note:** ALPS, analysis along the perivascular space; SBR, striatal binding ratio;

**Table S3** Partial correlation analysis of ALPS changes with MD of white matter.

| Regions | r | *p* |
| --- | --- | --- |
| L ATR | 0.322 | 0.007 |
| R ATR | 0.245 | 0.044 |
| L CST | 0.330 | 0.006 |
| R CST | 0.340 | 0.005 |
| L CG | 0.204 | 0.096 |
| L CG(HIP) | 0.277 | 0.022 |
| R CC | 0.372 | 0.002 |
| L IFO | 0.332 | 0.006 |
| R IFO | 0.106 | 0.388 |
| L ILF | 0.371 | 0.002 |
| L SLF | -0.103 | 0.406 |
| L UF | -0.228 | 0.063 |
| R UF | -0.068 | 0.583 |
| L SLF(T) | -0.198 | 0.108 |

**Note:** ATR, Anterior thalamic radiation; CC, corpus callosum; CG, Cingulum (cingulate gyrus); CG(HIP), Cingulum (hippocampus); CST, Corticospinal tract; IFO, Inferior fronto-occipital fasciculus; ILF, Inferior longitudinal fasciculus;L, left; R, right; SLF, Superior longitudinal fasciculus; SLF(T), Superior longitudinal fasciculus (temporal part) ;UF, Uncinate fasciculus;

**Table S4** Partial correlation analysis of ALPS changes with FA of white matter.

| Regions | r | *p* |
| --- | --- | --- |
| L ATR | -0.378 | 0.001 |
| R ATR | -0.387 | 0.001 |
| L CST | -0.306 | 0.011 |
| R CST | -0.334 | 0.005 |
| L CG | -0.410 | 0.001 |
| R CG | -0.338 | 0.005 |
| L CG(HIP) | -0.339 | 0.005 |
| R CG(HIP) | -0.294 | 0.015 |
| L CC | -0.328 | 0.006 |
| R CC | -0.438 | < 0.001 |
| L IFO | -0.276 | 0.023 |
| R IFO | -0.437 | < 0.001 |
| L ILF | -0.333 | 0.006 |
| R ILF | -0.455 | < 0.001 |
| L SLF | -0.435 | < 0.001 |
| R SLF | -0.255 | 0.036 |
| L UF | -0.262 | 0.031 |
| L SLF(T) | -0.402 | 0.001 |

**Note:** ATR, Anterior thalamic radiation; CG, Cingulum (cingulate gyrus); CG(HIP), Cingulum (hippocampus); CST, Corticospinal tract; IFO, Inferior fronto-occipital fasciculus; ILF, Inferior longitudinal fasciculus;L, left; R, right; SLF, Superior longitudinal fasciculus; SLF(T), Superior longitudinal fasciculus (temporal part) ;UF, Uncinate fasciculus;

**Table S5** Multivariate linear regression models for the association between changes in ALPS and MD of white matter.

| Regions | △ALPS Model characteristics | | | ALPS | | △MD | | Age | | Sex | |
| --- | --- | --- | --- | --- | --- | --- | --- | --- | --- | --- | --- |
|  | F | *p* | R2 | β | *p* | β | *p* | β | *p* | β | *p* |
| L ATR | 3.343 | 0.015 | 0.171 | -0.22 | 0.069 | 0.327 | 0.006 | -0.053 | 0.667 | 0.016 | 0.9 |
| R ATR | 2.380 | 0.061 | 0.128 | -0.22 | 0.077 | 0.255 | 0.041 | -0.05 | 0.702 | 0.051 | 0.697 |
| L CST | 3.579 | 0.011 | 0.181 | -0.232 | 0.055 | 0.34 | 0.004 | -0.061 | 0.618 | 0.031 | 0.805 |
| R CST | 3.302 | 0.016 | 0.169 | -0.185 | 0.129 | 0.327 | 0.007 | -0.062 | 0.619 | 0.004 | 0.978 |
| L CG | 2.103 | 0.090 | 0.115 | -0.232 | 0.065 | 0.224 | 0.073 | -0.059 | 0.651 | 0.015 | 0.910 |
| L CG(HIP) | 2.510 | 0.050 | 0.134 | -0.193 | 0.120 | 0.262 | 0.032 | -0.088 | 0.482 | 0.006 | 0.963 |
| R CC | 4.315 | 0.004 | 0.210 | -0.234 | 0.049 | 0.396 | 0.001 | -0.007 | 0.956 | -0.042 | 0.742 |
| L IFO | 3.348 | 0.015 | 0.171 | -0.204 | 0.092 | 0.325 | 0.006 | -0.072 | 0.558 | 0.009 | 0.947 |
| R IFO | 1.537 | 0.202 | 0.086 | -0.236 | 0.066 | 0.132 | 0.276 | -0.123 | 0.334 | 0.032 | 0.810 |
| L ILF | 4.161 | 0.005 | 0.204 | -0.220 | 0.064 | 0.378 | 0.002 | -0.037 | 0.761 | -0.002 | 0.986 |
| L SLF | 1.377 | 0.252 | 0.079 | -0.223 | 0.088 | -0.099 | 0.420 | -0.113 | 0.391 | 0.027 | 0.844 |
| L UF | 1.857 | 0.129 | 0.104 | -0.182 | 0.165 | -0.200 | 0.123 | -0.149 | 0.253 | 0.003 | 0.984 |
| R UF | 1.243 | 0.302 | 0.072 | -0.220 | 0.094 | -0.049 | 0.693 | -0.123 | 0.350 | 0.036 | 0.796 |
| L SLF(T) | 1.611 | 0.182 | 0.091 | -0.184 | 0.168 | -0.158 | 0.221 | -0.127 | 0.329 | 0.016 | 0.909 |

**Note:** ALPS, analysis along the perivascular space; ATR, Anterior thalamic radiation; CC, corpus callosum; CG, Cingulum (cingulate gyrus); CG(HIP), Cingulum (hippocampus); CST, Corticospinal tract; IFO, Inferior fronto-occipital fasciculus; ILF, Inferior longitudinal fasciculus;L, left; MD, mean diffusivity; R, right; SLF, Superior longitudinal fasciculus; SLF(T), Superior longitudinal fasciculus (temporal part) ;UF, Uncinate fasciculus;

**Table S6** Multivariate linear regression models for the association between changes in ALPS and FA of white matter.

| Regions | △ALPS Model characteristics | | | ALPS | | △FA | | Age | | SEX | |
| --- | --- | --- | --- | --- | --- | --- | --- | --- | --- | --- | --- |
|  | F | *p* | R2 | β | *p* | β | *p* | β | *p* | β | *p* |
| L ATR | 4.226 | 0.004 | 0.206 | -0.215 | 0.070 | -0.387 | 0.001 | -0.024 | 0.845 | -0.026 | 0.838 |
| R ATR | 4.257 | 0.004 | 0.208 | -0.199 | 0.093 | -0.382 | 0.001 | -0.047 | 0.698 | 0.009 | 0.944 |
| L CST | 2.964 | 0.026 | 0.154 | -0.203 | 0.098 | -0.309 | 0.013 | -0.061 | 0.627 | -0.043 | 0.750 |
| R CST | 2.853 | 0.031 | 0.149 | -0.134 | 0.290 | -0.297 | 0.016 | -0.099 | 0.425 | 0.048 | 0.709 |
| L CG | 4.870 | 0.002 | 0.231 | -0.212 | 0.069 | -0.432 | < 0.001 | 0.035 | 0.780 | -0.005 | 0.965 |
| R CG | 4.085 | 0.005 | 0.201 | -0.268 | 0.026 | -0.372 | 0.002 | -0.056 | 0.644 | 0.009 | 0.945 |
| L CG(HIP) | 3.660 | 0.009 | 0.184 | -0.226 | 0.061 | -0.344 | 0.004 | -0.069 | 0.571 | 0.010 | 0.936 |
| R CG(HIP) | 2.803 | 0.033 | 0.147 | -0.204 | 0.098 | -0.280 | 0.018 | -0.112 | 0.363 | 0.067 | 0.604 |
| L CC | 3.171 | 0.019 | 0.163 | -0.190 | 0.119 | -0.320 | 0.009 | -0.045 | 0.724 | 0.005 | 0.969 |
| R CC | 5.792 | < 0.001 | 0.263 | -0.235 | 0.040 | -0.447 | < 0.001 | -0.048 | 0.677 | 0.037 | 0.760 |
| L IFO | 2.764 | 0.035 | 0.145 | -0.224 | 0.069 | -0.281 | 0.019 | -0.093 | 0.456 | -0.005 | 0.968 |
| R IFO | 5.560 | 0.001 | 0.255 | -0.218 | 0.058 | -0.438 | < 0.001 | -0.051 | 0.660 | 0.014 | 0.909 |
| L ILF | 3.367 | 0.014 | 0.172 | -0.205 | 0.090 | -0.331 | 0.006 | -0.070 | 0.568 | -0.022 | 0.866 |
| R ILF | 5.790 | < 0.001 | 0.263 | -0.198 | 0.083 | -0.446 | < 0.001 | -0.080 | 0.484 | -0.013 | 0.916 |
| L SLF | 5.443 | 0.001 | 0.251 | -0.213 | 0.065 | -0.430 | < 0.001 | -0.081 | 0.487 | 0.001 | 0.994 |
| R SLF | 2.352 | 0.063 | 0.126 | -0.204 | 0.101 | -0.257 | 0.043 | -0.112 | 0.372 | -0.041 | 0.768 |
| L UF | 2.575 | 0.046 | 0.137 | -0.221 | 0.074 | -0.270 | 0.028 | -0.099 | 0.428 | -0.027 | 0.842 |
| L SLF(T) | 4.993 | 0.001 | 0.235 | -0.239 | 0.041 | -0.414 | < 0.001 | -0.091 | 0.434 | -0.031 | 0.805 |

**Note:** ALPS, analysis along the perivascular space; ATR, Anterior thalamic radiation; CG, Cingulum (cingulate gyrus); CG(HIP), Cingulum (hippocampus); CST, Corticospinal tract; FA, fractional anisotropy; IFO, Inferior fronto-occipital fasciculus; ILF, Inferior longitudinal fasciculus;L, left; R, right; SLF, Superior longitudinal fasciculus; SLF(T), Superior longitudinal fasciculus (temporal part) ;UF, Uncinate fasciculus;

Figure S1 The difference of FA (A) and MD (B) throughout the entire white matter (WM) skeleton in the cross-sectional study. TBSS results overlaid on the FA image. The green skeleton denotes the mean FA skeleton. Hot-colored voxels indicate white-matter regions showing significant group differences (FWE-TFCE corrected *p* < 0.01). Note: the hot-overlay intensities are shown for visualization after tbss_fill thickening and should not be interpreted as quantitative t-statistics or (1−*p*) values.


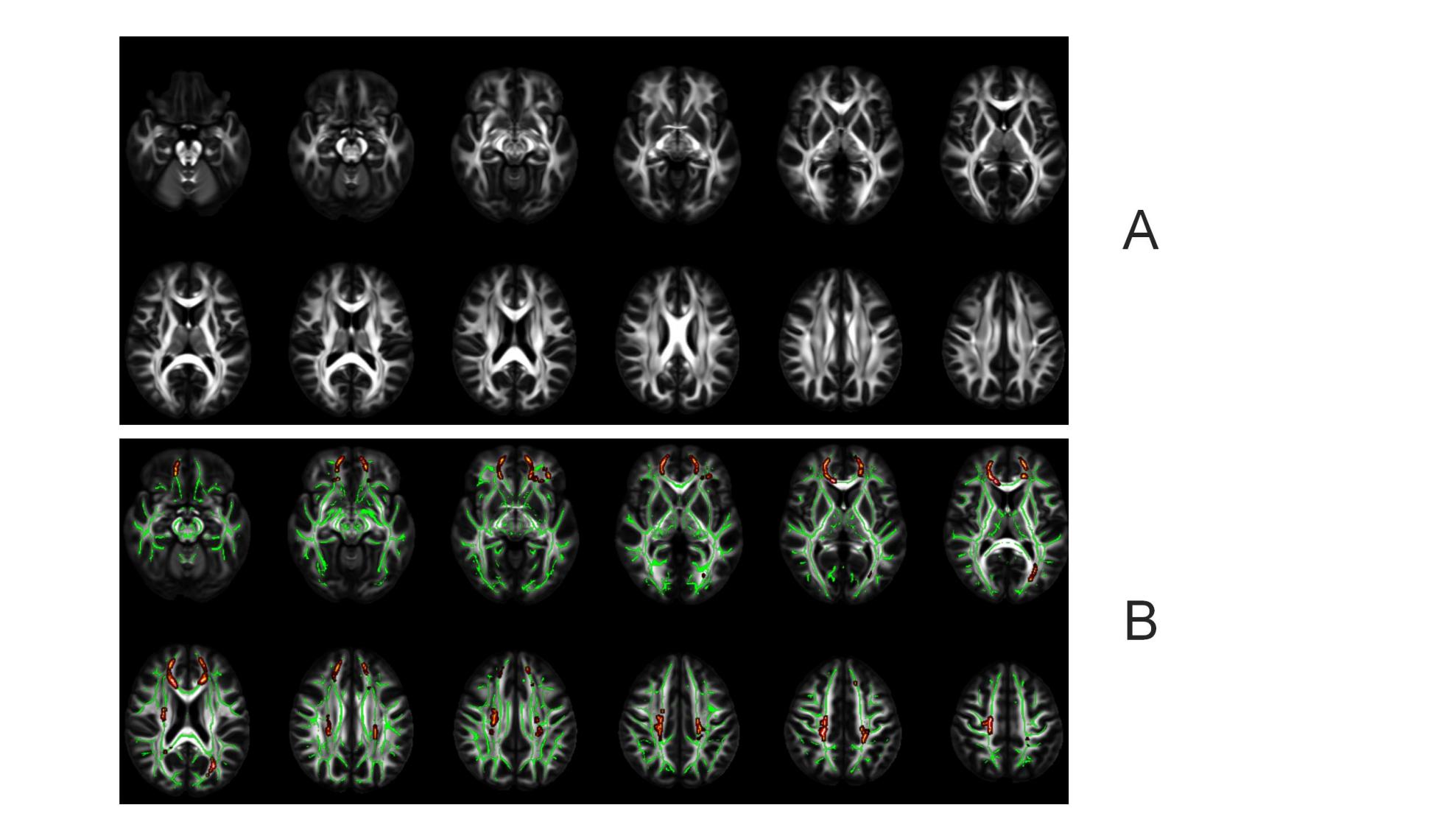


Figure S2 The difference of FA (A) and MD (B) throughout the entire white matter (WM) skeleton in the longitudinal study. TBSS results overlaid on the FA image. The green skeleton denotes the mean FA skeleton. Hot-colored voxels indicate white-matter regions showing significant group differences (FWE-TFCE corrected *p* < 0.01). Note: the hot-overlay intensities are shown for visualization after tbss_fill thickening and should not be interpreted as quantitative t-statistics or (1−*p*) values.


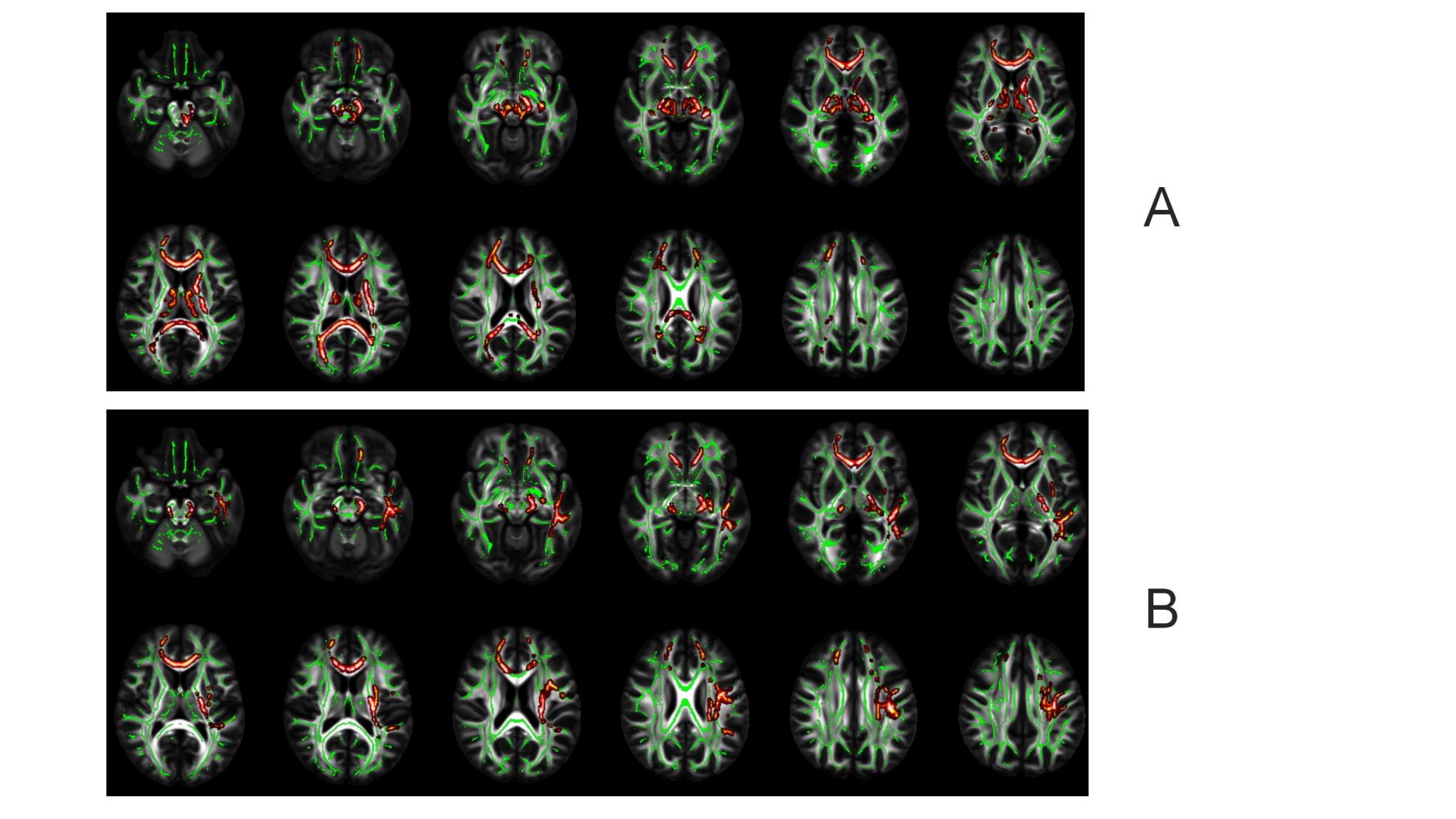

Supplement: Supplementary file 1 — Data S1: Supporting Information. [file HBM-47-e70477-s001.docx]
